# Spinal integration of hot and cold nociceptive stimuli by wide-dynamic-range neurons in anesthetized adult rats

**DOI:** 10.1097/PR9.0000000000000983

**Published:** 2021-12-16

**Authors:** Clémence Gieré, Meggane Melchior, André Dufour, Pierrick Poisbeau

**Affiliations:** a Centre National de la Recherche Scientifique, University of Strasbourg, Institut des Neurosciences Cellulaires et Intégratives, Strasbourg, France; b Centre National de la Recherche Scientifique, University of Strasbourg, Laboratoire de Neurosciences Cognitives et Adaptatives, Strasbourg, France

**Keywords:** Nociception, In vivo electrophysiology, Thermosensitivity, Pain, Cutaneous nociceptors

## Abstract

**Introduction:**

Early neuronal processing of thermal noxious information relies mostly on molecular detectors of the transient receptor potential family expressed by specific subpopulation of sensory neurons. This information may converge to second-order wide-dynamic-range (WDR) neurons located in the deep layer of the dorsal horn of the spinal cord.

**Method:**

Using a micro-Peltier thermode thermal contact stimulator II delivering various cold and hot noxious stimulations, we have characterized the extracellular electrophysiological responses of mechanosensitive WDR neurons in anesthetized adult male and female Wistar rats.

**Results:**

Most of the WDR neurons were activated after hot and cold noxious stimulations, at mean temperature thresholds corresponding to 43 and 20°C, respectively. If the production of action potential was not different in frequency between the 2 thermal modalities, the latency to observe the first action potential was significantly different (cold: 212 ms; hot: 490 ms, unpaired Student *t*-test: t = 8.041; *df* = 32; *P* < 0.0001), suggesting that different fiber types and circuits were involved. The temporal summation was also different because no facilitation was seen for cold noxious stimulations contrary to hot noxious ones.

**Conclusion:**

Altogether, this study helps better understand how short-lasting and long-lasting hot or cold noxious stimuli are integrated by mechanosensitive WDR neurons. In our experimental conditions, we found WDR neurons to be nociceptive specific for C-fiber–mediated hot stimuli. We also found that cold nonnoxious and noxious information, triggered at glabrous skin areas, are likely taken in charge by A-type sensory neurons. This study will be helpful to establish working hypothesis explaining the thermal pain symptoms displayed by animal models and patients in a translational extent.

## Abstract 2

Supplemental Digital Content is Available in the Text. Mechanosensitive wide-dynamic-range spinal neurons differentially integrate thermal noxious information applied using a hot or cold micro-Peltier device.

## 1. Introduction

In the nervous system, interoceptive and exteroceptive nociceptive stimuli are detected and coded by sensory neurons innervating tissues and organs. These stimuli are of various nature and intensity, such as mechanical deformation, heat, cold, or irritation caused by chemicals. Regarding thermal modalities, the molecular detection is mostly performed by the transient receptor potential (TRP) ion channel superfamily.^5^ If the gating mechanism is still unclear, it seems that nonselective cationic TRP ion channels are activated thanks to an allosteric coupling between protein modules, respectively, sensitive to temperatures and voltage changes.^24^ This proposal also explains well the voltage-dependent influences on the TRP channel function and temperature thresholds. The thermal sensitivity of these TRPs in the skin is quite exceptional because they are capable of coding hot or cold temperature changes of less than 1°C.^30^ Since the discovery of the transient receptor potential vanilloid type 1 (TRPV1), which is activated by temperature higher than 42°C and capsaicin, an active ingredient in chili,^6^ several thermosensitive TRPs have been characterized in mammalian sensory neurons. In the 7 families of TRPs, thermosensitive channels are found in the ankyrin subtype (TRPA1), vanilloid set (TRPV1-4), canonical type (TRPC5), and melastatin subtype (TRPM2-5 and TRPM8).^5^

Heat threshold triggering activation of TRPV1, through temperature sensor domains which include the distal half of the C terminus,^38^ is found at temperature values around 42°C.^6^ TRPV1 is expressed by half of the dorsal root ganglion sensory neurons and by 75% of small diameter unmyelinated C-type neurons which represent most of the nociceptive-specific neurons.^13^ Transient receptor potential vanilloid type 2 are also activated by noxious hot temperatures exceeding 52°C but are mostly expressed by some Aδ-type sensory neurons.^2^ In comparison with TRPV1 and TRPV2, TRPV3 and TRPV4 seem to be involved in the thermal detection of nonnoxious hot temperature because their activation can be seen in the nonnoxious range, starting at 20 and 32°C for TRPV3 and TRPV4, respectively.^12^ Although TRPV3 can be involved in the detection of temperature around 40°C, it is unlikely that TRPV3 and TRPV4 play a major role in thermal nociception.^20^

The nociceptive cold threshold in humans can be reached when temperatures are generally below 15°C.^10^ Two subpopulations of sensory neurons seem to be involved. The first subpopulation is made of nociceptors expressing the type 8 melastatin TRP channel (TRPM8). They are activated by temperatures below 25°C or by natural substances inducing a sensation of cold, such as menthol or icilin.^27^ The second neuronal population is expressing the type 1 ankyrin TRP channel (TRPA1). They also express TRPV1 and are activated at temperatures below 17°C or by chemicals inducing a sensation of burning like with allyl isothiocyanate, the active component of mustard, horseradish, and wasabi.^33^ This TRPA1–TRPV1 coexpression could explain the feeling of burning induced by noxious cold stimuli.

Although the mechanisms underlying thermal sensitivity have been greatly studied in primary sensory neurons, the integration of such information in the spinal cord or in supraspinal centers is far less understood. The local microcircuits processing this thermal information in the superficial dorsal horn of the spinal cord are still under investigation.^21^ One of the first study that attempted to establish a characterization of thermal response in the spinal cord proposed a classification of neurons dependent on their activity in response to mechanical, hot, and cold stimuli; class 1 units were responsive to innocuous stimuli but not nociceptive, class 2 units responded to both innocuous and nociceptive stimuli, and class 3 units only to nociceptive stimuli. Only class 1 and class 2 units were heat responsive and were further divided into (1) warming neurons activated by temperature below 42.5°C, (2) warming or noxious units activated by temperature below 42.5°C but with peak activity at higher temperatures, and (3) noxious units activated above 42.5°C. All heat responsive units were also activated by cooling, but a specific characterization of the cold response was not conducted.^29^ In this study, thermal responsive neurons were located in laminae I, IV, and V. Early c-fos studies also investigated the spinal integration of cold or hot noxious stimulation. They showed that after noxious heat, 57% to 69% of lamina I NK1 receptor–expressing projection neurons expressed c-fos and that the proportion did not differ between the different morphological groups of neurons (pyramidal, fusiform, and multipolar cell). However, noxious cold activated mostly multipolar neurons and only a small portion of fusiform or pyramidal cells.^34^ A previous study on cats also reported that pyramidal cells responsed to innocuous cooling, fusiform and multipolar cells responsed to noxious heat, and only multipolar cells responded to noxious cooling.^18^ The intensity of c-fos expression in the spinal cord in response to noxious cold was showed to be intensity dependent and was absent or very small for temperatures above −10°C.^1^ Dado et al. recorded spinal neurons of the spinothalamic tract and showed that 86% responded to noxious heat and 29% to cooling with a threshold at 29.1°, with a majority responding to both noxious and innoxious cold.^7^ Concerning wide-dynamic-range (WDR) neurons, their response to noxious heat and cold was previously investigated by Khasabov et al.,^23^ who showed that 86% of WDR neurons responded to both noxious heat and cold and 14% only to heat, whereas 61% of high-threshold nociceptive-specific neurons responded to both noxious modalities and 32% and 7% only to heat and cold, respectively. They also showed that the mean cold threshold of WDR neurons was higher that the one of high-threshold neurons (∼15 vs −0.6°).

A recent in vivo calcium imaging study has been able to provide further insights into the coding and processing of thermal stimuli in the superficial layers.^31,36^ These data show a functional difference in the processing of warming and cooling, as well as at the response to noxious stimuli. Cold responses peaked during the transient phase of the stimuli and then rapidly adapted, whereas hot responses persisted during the whole stimulus. These results seem to indicate that processing of the cold response does not necessarily reflect the temperature of the skin but rather its change of temperature, whereas the coding of heat is absolute with little adaptation from the transducting fibers.

In this study, we have recorded WDR neurons in the deep dorsal horn layers of the spinal cord (400–1000 µm) to characterize their role in the processing of nociceptive and nonnociceptive thermal information. More precisely, we recorded mechanoresponsive WDR neurons and characterized their integrative properties after thermal stimulation of peripheral receptive field on the hind paw. Based on data from the literature,^35^ we expected that these neurons would respond to a convergence of both innocuous and noxious information, from mechanical and thermal modalities. So far, characterization of their integrative role for different modalities has been a challenge. Thermal stimulations used were often too slow (water jet, water bath, ice, or conducting metals) to evaluate thermal thresholds (skin inertia bias) and not properly controlling the receptor field local temperature.^15^ Here, we used stimulation (300°C/second) with cold or hot noxious temperatures, with various duration (short pulses, ramps, or sustained stimuli) while controlling the surface skin temperature, granting a greater control over the stimulation parameters. Thanks to this novel device, these stimulations were applied on a small receptive field while recording WDR neurons in the dorsal horn of the spinal cord using extracellular recording in isoflurane-anesthetized adult rats. Apart from testing the hypothesis that cold and hot information from a given receptive field converge to WDR neurons, we assessed the thermonociceptive-specific role of WDR neurons and the relative contribution of C-type and A-type fibers in this processing.

## 2. Material and methods

### 2.1. Animals

Adult male and female Wistar rats (aged P45-P60) were used for this study. No differences were found between males and females, so data were pooled (see supplementary Fig. 1, available at http://links.lww.com/PR9/A144). Rats were housed in littered cages, in a temperature-controlled room (22°C) at a hygrometry of 45 ± 5%, under a 12-hour light–dark cycle (lights on at 7 am), with ad libitum access to food and water. The experiments were performed after a week of acclimatization in the animal facility. All experiments conducted were approved by the regional ethical committee regarding animal experimentation (APAFIS #201706131614598) and complied to the ARRIVE guidelines.

### 2.2. *In vivo* extracellular single-unit electrophysiological recordings

We used an in vivo preparation for the extracellular single-unit recording, as previously described.^22^ Rats were anesthetized with isoflurane 4% pushed by pressured air at a flow rate of 700 mL/min (Ventoflurane, Vibrac, Carros, France) and placed onto a stereotaxic frame equipped with a nasal mask to maintain anesthesia and with an electrical warming blanket to keep a steady body temperature (Ugo Basile, Rodent Warmer X1, Gemonio, Italy). Isoflurane was adjusted to 3% for the ensuing surgery which started when the rat was areflexic. A laminectomy was performed at the lumbar level (L3-L5). With the cervical and sacral vertebrae firmly held, the meninges were carefully removed, the exposed segment of spinal cord was covered with an isolating mineral oil to prevent dehydration, and the anesthesia was adjusted to 1.5% for the recordings. At the end of the experiment, animals were killed with an overdose of anesthesia (isoflurane 5%).

Single-unit extracellular recordings were made from WDR neurons located in the deep layers of the dorsal horn (400–1000 µm) with a stainless steel microelectrode (FHC, Stainless Steel, UE FK1, Bowdoin) lowered into the medial part of the spinal cord by an electrical micromanipulator (Scientifica, IVM 1000, Uckfield, United Kingdom). Variations of potential were amplified (DAM80, AC Differential Amplifier, WPI, Aston, United Kingdom) and filtered for frequency superior to 3 kHz and inferior to 300 Hz. Parasite frequencies were suppressed using a noise eliminator (HumBug 50/60 Hz Noise Eliminator, WPI, Aston, United Kingdom), and the resulting signal was digitized with an acquisition frequency of 10 kHz (Power 1401, CED, Cambridge, United Kingdom) and processed by the Spike2 software (CED, Cambridge, England). In the case of multicellular recordings, unitary responses were isolated thanks to the Spike Sorting Analysis Module of Spike2 software.

Neurons were selected on the basis of their response to innocuous (light brush applied on the receptor field and light pressure applied with the finger) and nociceptive (pinching with a pair of forceps) mechanical stimuli and were then tested with thermal stimuli. This not only allowed their identification as convergent second-order neurons but also permitted to precisely define the receptor field on the animal's paw.

After a single thermal stimulation of the peripheral receptive field, WDR neuron frequency of extracellularly recorded action potential (AP) discharges was measured during 2 seconds, corresponding to the duration of the stereotypical bursting discharge of hot-sensitive neurons. To evaluate the activation threshold temperature of stimulated sensory fibers, the latency to observe the first AP was measured in response to a slow-heating or slow-cooling ramp, from the neutral temperature (30°C) to 52 or 0°C, for noxious hot and cold modalities, respectively. The heating ramp speed was of 3°C/second, whereas the cooling ramp speed was of 4°C/second for a maximum stimulation duration of 7.5 seconds. The frequency of discharge for the duration of the stimulation was also measured. When spontaneous activity was already present, the mean baseline frequency before stimulation was removed from the poststimulation data.

### 2.3. Wind-up protocol

Spinal WDR neurons recorded in the deep layer of the dorsal horn are known to reproduce with fidelity the «wind-up» phenomenon observed when repeated electrical stimulation (1 Hz) are applied on C fibers. A similar protocol was used here for hot and cold nociceptive stimuli (30 single stimuli, 1 Hz) to assess the C fibers response to a sustained thermal stimulation. The frequency of response of the neuron was calculated in Hz for the 30 seconds of stimulation. This protocol was chosen to study the desensitization of thermal responses as well as to potentially observe a facilitation of the response such as the «wind-up» response resulting from repeated electrical stimulation.

### 2.4. Stimulation thermode

**Figure 1.**
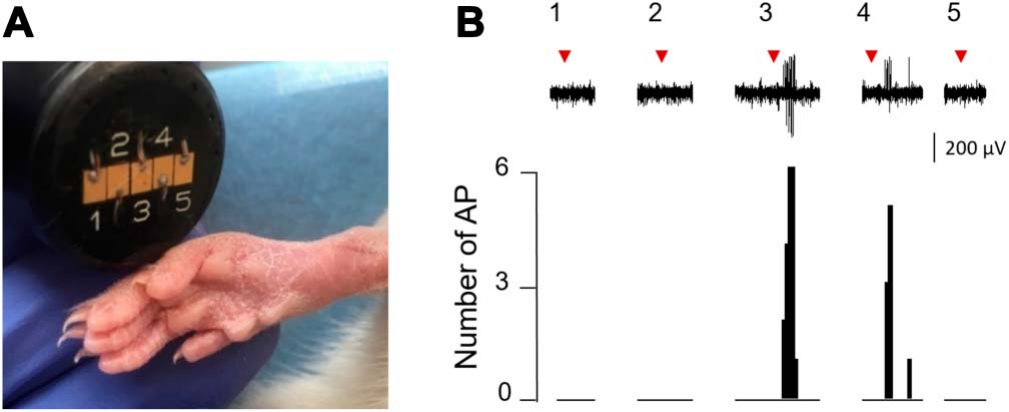
Thermode micro-Peltier TCS II characteristics and its interest while recording spinal wide dynamic range (WDR) neurons in vivo. (A) Image illustrating the size of the stimulation zones (8 mm^2^) with respect to the adult rat hind paw size. (B) Representative extracellular unit recording of a WDR neuron in response to nociceptive hot (52°C, heating speed 300 °C/s; duration: 1 second) delivered by one of the 5 stimulation zone (numbered 1–5). The corresponding receptor field for this WDR neuron overlaps on the third and fourth stimulated areas. AP, action potential; TCS, thermal contact stimulator.

Thermal stimulations were applied using a thermal contact stimulator (TCS II, Thermal Cutaneous Stimulator, QST.Lab, Strasbourg, France), allowing the stimulation of the cutaneous surface (minimum 8 mm^2^) with temperature ranging from 0 to 60°C. The heating or cooling rate could be adjusted from 0.1 to 300°C/second.^11^ The device we used provides 5 independent stimulation areas of 8 mm^2^ granting the possibility to perform very precise stimulation to a slower heating or cooling ramp (Fig. 1). As soon as the hind paw glabrous skin receptive field was identified for given WDR neurons, we placed the thermode on the site until the end of the recording. The different stimulatory procedures are given in Table 1.

**Table 1:** Summary table of the stimulation protocols used in the study.

	Hot	Cold	Measured parameters
Single stimulation	52°C Speed: 300°C/second Duration: 250, 500, 750, and 1000 ms	0°C Speed: 300°C/second Duration: 250, 500, 750, and 1000 ms	Latency to first action potential Frequency of discharge
Slow ramp	30->52°C Speed: 3°C/second Duration: 7.5 seconds	30-> 0°C Speed: 3°C/second Duration: 7.5 seconds	Activation threshold Frequency of discharge
Sustained stimulation	30 × Single stimulation Frequency: 1Hz	30 × Single stimulation Frequency: 1Hz	Frequency of discharge
Hot and cold response curve	Temperatures: 30, 35, 40, 45, and 52°C Duration: 5 seconds	Temperatures: 20, 15, 10, 5, and 0°C Duration: 5 seconds	Frequency of discharge

### 2.5. Statistical analysis

Graphs and statistics were generated with GraphPad Prism 6 (GraphPad software Inc, California). Data are expressed in mean ± SEM. We used parametric tests (unpaired Student *t* test) when data were normally distributed. Normality was verified with the D'Agostino–Pearson omnibus normality test. If not, nonparametric (Mann–Whitney) statistical tests were used. A *P* value < 0.05 was considered statistically significant.

## 3. Results

### 3.1. Identification of thermal sensitive wide-dynamic-range neurons

**Figure 2.**
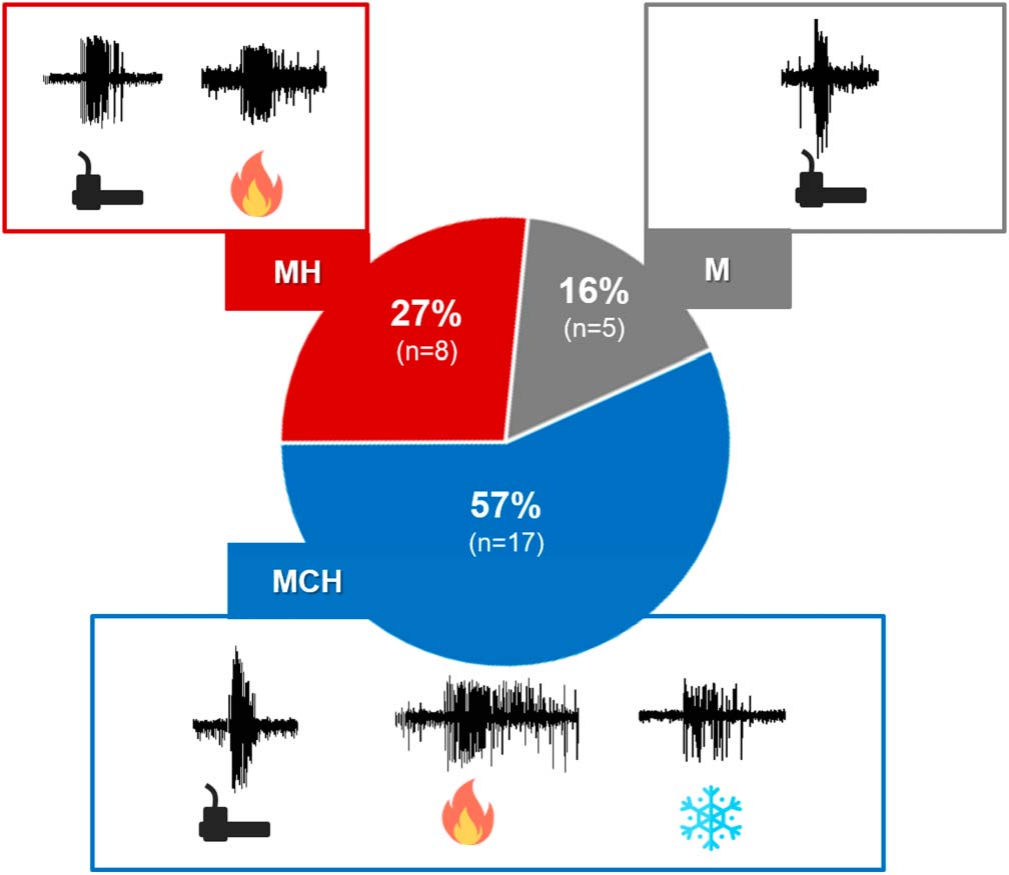
Proportion of wide dynamic range neurons responsive to hot, cold, and mechanical stimuli. M, mechano; MCH, mechano-heat-cold; MH, mechano-heat.

A total of 37 neurons were recorded. Among them, 5 nonnociceptive neurons and 2 nociceptive-specific neurons were excluded from the analysis. Of the remaining WDR neurons responding to mechanical stimuli (n = 30), 83% were also activated by noxious heat (MH: mechano-heat neurons, n = 25) and 57% to cold and heat (MHC: mechano-heat-cold neurons n = 17) (Fig. 2). All neurons that responded to cold stimuli also responded to heat. Notably, very little to no basal activity as well as no response to nonnociceptive thermal stimulation was recorded.

### 3.2. Single thermal stimulation

**Figure 3.**
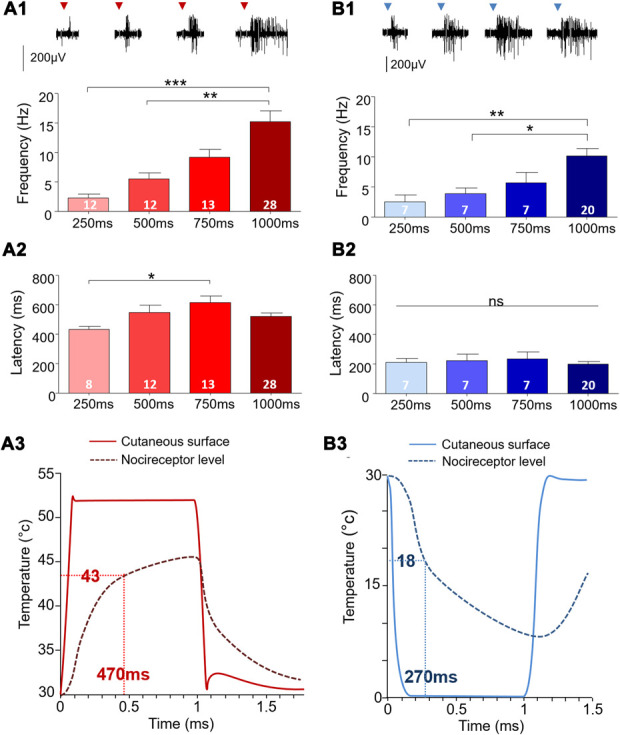
Characterization of wide dynamic range neurons extracellular action potential discharges after a single thermal stimulation (ramp 300°C/second) with various durations. Representative examples of a wide dynamic range neuron response to a hot (A1-2) or a cold (B1-2) paw stimulation with a duration of 250, 500, 750, and 1000 ms. After analysis of variance (refer to text), Tukey post hoc tests were performed for multiple comparison (code: ***, *P* < 0.0001; **, *P* < 0.01; *, *P* < 0.05; ns: not significant. (A3-B3) Model predicting the temperature reached at the cutaneous surface and at the nociceptor level (localized around 100 µm below the skin's surface) during a single hot (A3) stimulation of 52°C or a single cold (B3) stimulation of 0°C (ramp 300 °C/s) during 1000 ms. The number of recorded cells is given in the graphs.

In the first part of the work, a single stimulation was applied for a duration of 250, 500, 750, and 1000 ms with an ultrafast heating at 52°C or cooling at 0°C (ramp of 300°C/second), from the baseline temperature set at 30°C (Fig. 3). As shown in the figure, we observed a stimulation duration-dependent increase of AP discharge frequency (hot: analysis of variance [ANOVA], F_(3,68)_ = 9.87; *P* < 0.0001; cold: ANOVA, F_(3,33)_ = 5.95, *P* = 0.0023). For latencies, a statistical difference was only found in the case of hot stimulation (hot; ANOVA, F_(3,57)_ = 3.22; *P* = 0.029; cold: ANOVA, F_(3,33)_ = 0.3, *P* = 0.83). Using the parameters given in a previously published article,^14^ the model describing temperature changes at the cutaneous surface and at the nociceptor depth (150 µm) is given in Figure 3. It indicates the theoretical duration required to reach noxious temperature for heat (43°C) and cold (18°C) while using a contact thermode and our stimulation protocol (Fig. 3). We can see that the theoretical time to reach the hot nociceptive threshold (43°C) at the receptors (ie, at 150 µm below the skin surface) is 470 ms while the time to reach the equivalent cold delta from baseline temperature (18°C) is 270 ms. This difference in latency is mainly due to the temperature set point imposed on the probe, ie, 52 vs 0°C, ie, a difference of 8°C between the 2 conditions, starting from the base temperature of 30°C.

**Figure 4.**
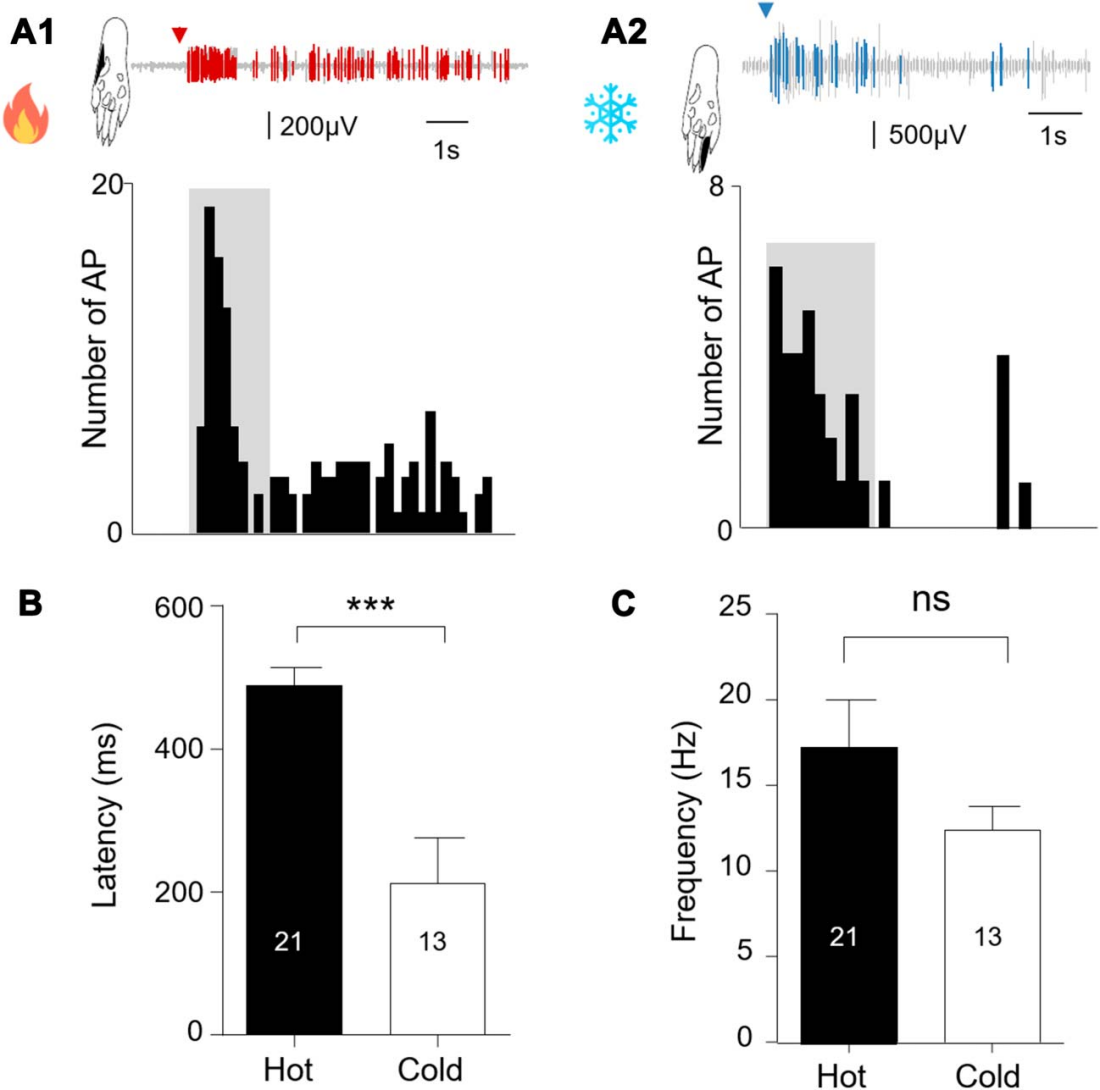
Extracellular recording of wide dynamic range neurons in the dorsal horn of the spinal cord after a single hot (A1) or cold (A2) stimulation (ramp 300°C/second, duration of 1000 ms). Red (A1) and blue (A2) segments within the recording trace correspond to the firing of a single neuron after spike sorting analysis. The black area in the rat hind paw drawing gives the localization of the receptive field stimulated. Histograms gives the number of AP detected per bins of 200 ms. Graphs showing the mean latency to observe the first AP after a hot or cold single stimulation (B) and the corresponding mean frequency (C). Statistical code for the Student *t*-test: *P* < 0.0001 (***); ns: nonsignificant. The number of recorded cells is given in the graphs. AP, action potential.

When comparing the response between hot and cold stimuli for a 1000-ms stimulation, we observed that the latency to the first emitted AP was significantly higher (unpaired Student *t*-test: t = 8.041; *df* = 32; *P* < 0.0001) for the hot (490 ± 24 ms; n = 21) than the cold stimulation (212 ± 18 ms; n = 13; Fig. 4). The mean frequency of AP discharge, however, was not different between the 2 modalities (hot = 17.2 ± 2.8 Hz; cold = 12.4 ± 1.4 Hz; unpaired Student *t*-test: t = 0.1283; *df* = 32; *P* = 0.2087; Fig. 4).

### 3.3. Slow ramp stimulation

**Figure 5.**
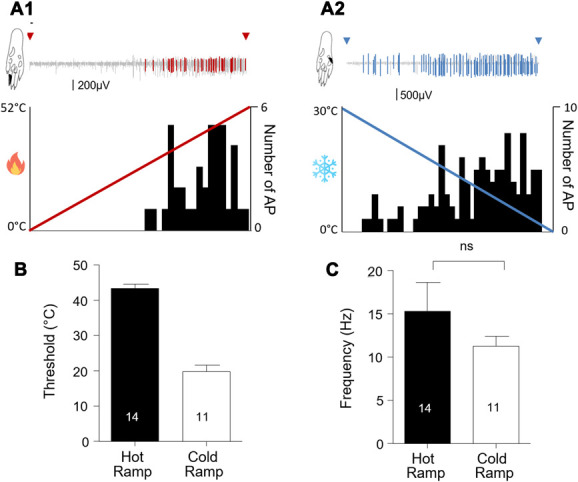
Electrophysiological responses of wide dynamic range spinal neurons after stimulation of the paw receptive field with a slow heating (3°C/second) or cooling (4°C/second) temperature ramp (duration: 7.5 seconds). (A1, A2) Representative traces recorded after a slow hot or cold ramp. Red (A1) and blue (A2) segments within the recording trace correspond to the firing of a single neuron after spike sorting analysis. The black surface on the paw indicates the receptive field stimulated for this response. Histograms show the number of AP per bins of 200 ms. (B) Mean temperature threshold to observe the first AP after hot or cold stimulation of the peripheral receptive field. (C) Mean frequency of AP discharge (Hz) while analyzing the whole period of firing. The number of recorded cells is given in the columns. Statistical code for the Student *t*-test, ns: nonsignificant. AP, action potential.

Representative responses of WDR neurons to heat and cold stimulations are shown in Figure 4A. As seen in Figure 4B, the threshold for hot stimulation was of 43.4 ± 1.2°C (n = 14) and for cold stimulation was of 19.8 ± 1.8°C (n = 11). Similarly to single stimuli, the mean frequency of AP firing did not differ (Student *t*-test: t = 1.013; *df* = 23; *P* = 0.3214) between both modalities (hot = 15.3 ± 3.4 Hz; n = 14; cold = 11.3 ± 1.2 Hz; n = 11; Fig. 5).

### 3.4. Sustained stimulation

**Figure 6.**
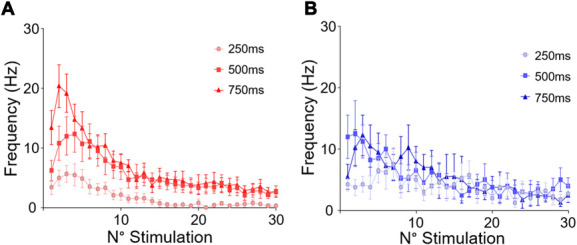
Adaptation of the wide dynamic range neuronal discharge after an iterative stimulation (30 stimulation applied at 1 Hz) of the paw receptive field for noxious hot (52°C, A) or cold (0°C, B). Several stimulation durations were tested.

We then assessed the electrophysiological properties of thermoresponsive WDR neurons while coding for repeated hot and cold stimulations (30 stimulations at 1 Hz) of various durations (Fig. 6). For repeated hot stimulation, we observed a slight increase in AP firing within the first few seconds (refer to the first 3 stimulations in the figure) followed by a progressive decrease in the frequency during the remaining part of the stimulation protocol.

For cold stimuli, no such facilitation could be observed, but the frequency of discharge did show a slight decrease until the end of the protocol.

**Figure 7.**
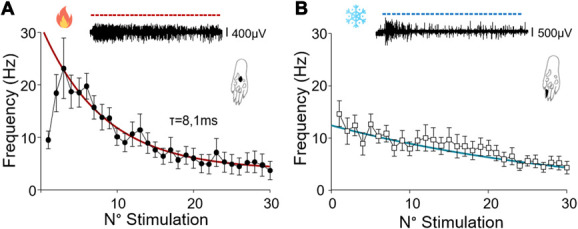
Wide dynamic range neurons coding after 30 stimulations of the paw receptive field with 1000 ms thermal noxious stimulations at 1 Hz. Continuous stimulation's response to 30 repeated hot (52°C; n = 10) or cold (0°C; n = 6) stimuli (300 °C/s, duration 1000 ms, frequency 1 Hz). Representative traces (top panels) showing the action potential discharge pattern for 30 stimulations at 52°C (A) and 0°C (B). The number of action potential is also given in the histogram for a 200-ms period. Bottom panels gives the frequency per stimulation period. Lines indicates the exponential fit which perfectly adjusted the reduction of the frequency with time.

As illustrated in Figure 7, in response to hot stimulations, a short-lasting facilitation of the WDR response can be easily observed during the first 3 stimulations. This could also be seen in the case of continuous stimulation (duration 1000 ms). Action potential discharge frequency increased from 9.5 ± 1.7 Hz (stim 1) to 23.1 ± 5.8 Hz (stim 3). After these first 3 seconds, a progressive decrease was observed which could be fitted with an exponential decay constant (τ) of 8.12 ms. The mean frequency at the end of the 30 stimulation protocols was strongly reduced and of 3.7 ± 1.7 Hz. As mentioned above, no facilitation could be observed for the cold stimulation, even if this stimulation was continuous (1000 ms duration). The frequency was maximal at the beginning of the protocol (17.1 ± 3.3 Hz) but slightly decreased with time. The mean frequency was of 4.2 ± 1.1 Hz at the end of the protocol (Fig. 7).

### 3.5. Hot and cold response curve

**Figure 8.**
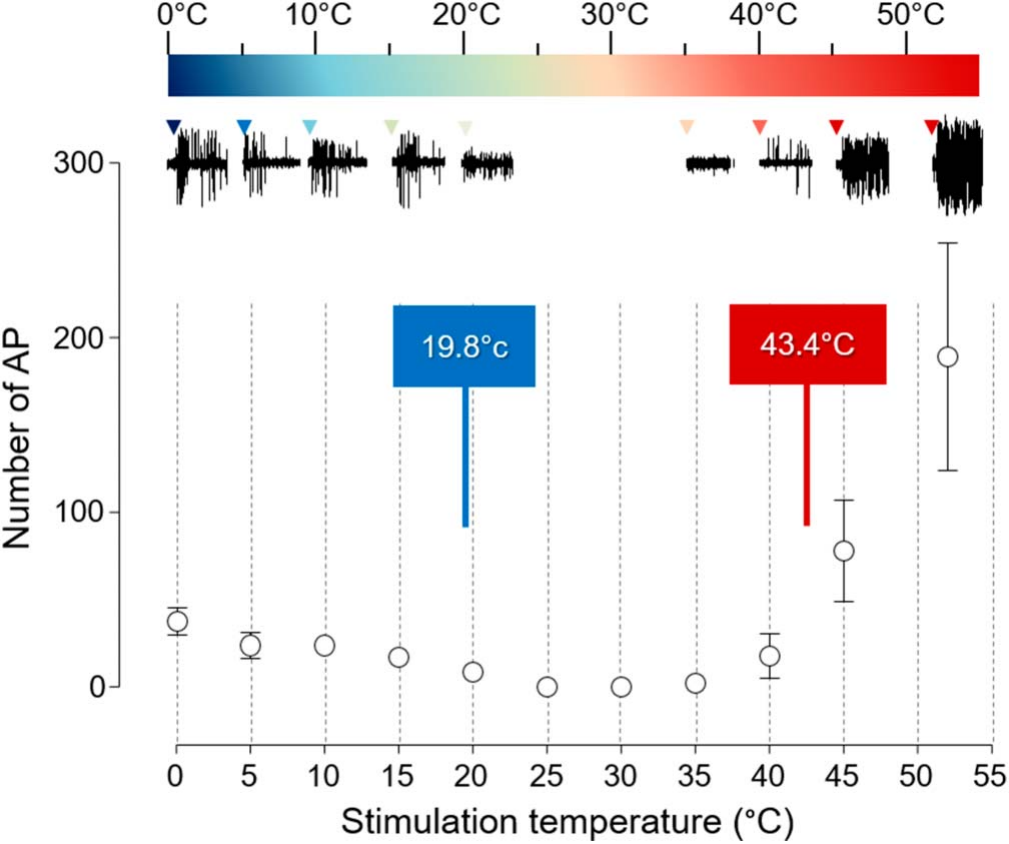
Specific processing of mechano-heat-cold wide dynamic range neurons (n = 7). The mean number of action potential (action potential (AP)) is indicated for each temperature used to stimulate the paw receptive field (300 °C/s; duration 5000 ms). The threshold activation temperature for both hot and cold is also represented.

Figure 7 illustrates the responses seen in mechano-heat-cold WDR neurons (MHC, n = 7) (Fig. 8). Here, we applied a single stimulation (300°C/second, duration 5000 ms) from 0 to 52°C (increments of 5°C). Interestingly, these MHC neurons had different coding frequency depending on the cold and hot stimuli. The action potential number peaked at 25 for temperatures below 15°C, whereas more than 100 APs could be seen if noxious hot stimulations were applied (>40°C).

## 4. Discussion

In this work, spinal WDR neurons responding to mechanical stimulations were recorded to further characterize their processing capacities after sensory spinal transmission of cold and hot noxious information. Most of the WDR neurons (83%) recorded were activated when noxious hot stimuli were applied to the hind paw receptive field. Surprisingly, we found that WDR neurons seem to respond in a nociceptive-specific manner to hot stimuli because they started to fire action potential at a threshold of about 42° (ie, the usual nociceptive threshold for heat). Half of them (53%) also processed cold messages, both noxious and nonnoxious. Using a single stimulus, we found that the latency to observe the first AP after the onset of the stimulation was much faster for cold (about 210 ms) than for hot noxious temperatures (about 490 ms). No differences could be seen in the mean frequency of occurrence of extracellular AP generated by WDR after cold or hot noxious stimulations (range between 10 and 20 Hz). When analyzing the coding properties about iterative stimulations at 1 Hz, only hot noxious processing was associated with an immediate short-lasting wind-up response. This result is in good agreement with the long latencies measured before the appearance of the first AP and the likelihood that these hot noxious messages are carried by unmyelinated C fibers. Our results also suggest that cold messages may use other sensory neurons such as those from the Aδ-type family. It is also to be noted that our study was made on glabrous skin which could explain differences in the threshold of proportion of thermoresponsive neurons compared with other studies made on hairy skin.^32^

Thermal thresholds obtained during the slow ramp protocols and the modelisation of the stimuli are in good agreement with those found in the literature, ie, around 42°C for heat and below 15°C for cold.^6,10^ Therefore, it seems that slow and progressive heating or cooling of the cutaneous surface diminished the influence of the skin's inertia observed during the stimulation. These results highlight the interest of using this heating or cooling thermode in preclinical studies as well as in clinical ones. It is currently in use in several research laboratories for quantitative sensory testing or to trigger extracellular receptor potential from the cortex.^40^ It is to be noted that the cold threshold obtained in this study comes from the mean of recorded threshold which varied from 25°C to 10°C. This apparent heterogeneity is in accordance with the literature which describes a large range of sensitivity in cold-responsive nociceptors.^2^

If an electrical stimulation of the peripheral receptive field is used (ie, to induce an immediate depolarization of primary afferent fibers without stimulating the nociceptive molecular detectors), fast conducting A-type sensory axons will activate more rapidly the second-order WDR neurons than the slow-conducting C-type sensory axons [see for example Ref. 22]. Regarding their conduction velocities and the appearance of the first AP discharge, it seems likely that cold information reaching the WDR neurons used Aδ fibers to reach spinal WDR neurons of the deep dorsal horn. The recorded response may also depend on other factors, including the thermode heating or cooling time or the thermal inertia of the skin. Indeed, our analysis of the hot and cold temperature ramps of the probe as well as the thermic diffusion properties of the skin (Fig. 2) shows that there is a significant difference in latency to reach the same temperature deltas from baseline between hot and cold stimuli. AP generation after the corresponding nociceptive receptor activation (ie, transduction time) may also be a nonnegligible factor.

As mentioned above, the latency of the first AP emitted after cold noxious stimuli (≈221 ms) does not seem consistent with an information transmitted by slow-conducting unmyelinated C fibers, which backs the hypothesis that the fast response observed in recorded spinal WDR neurons would correspond to a convergence of information carried by Aδ fibers. Moreover, Aδ fibers are known to be adaptive,^26^ meaning that they rapidly cease to fire on a sustained stimulation. This hypothesis is well illustrated by the sustained stimulation protocol showing the adaptive nature of the cold response with the rapid decrease of AP firing within the first few seconds. This result is also in good agreement with previous works conducted on cold-sensitive fibers in the monkey,^9^ demonstrating a short latency to the cold response, followed by a fast decrease of firing as well as an inhibition of the response after repeated stimulation.

The results from the various hot stimulation protocols give less clear-cut conclusions, although the latency would argue in favor of a peripheral detection and transmission by C fibers. The observed latency (≈441 ms) is in accordance with the work of Xu et al.^39^ who modeled a response by C nociceptors to heat with a latency of 600 ms. The discrepancy between our value and theirs could come from the type of metal used to heat the skin which implies a different coefficient of thermal conduction.^37^ Moreover, the presence of postdischarge, characteristic of C fibers,^28^ was often observed after stimuli of 52°C, but not at 0°C (as illustrated in Fig. 3). The hypothesis of the perception of nociceptive heat mostly by C fibers is also consistent with (1) the modelisation of the tail-flick reflex^3,8^ which does not include the involvement of Aδ fibers and ii) the observation that the glabrous skin—as in our study—seems to be less innervated by heat-responsive Aδ fibers.^4,17^ Another explanation could be that the recorded neurons in the deep layer of the spinal dorsal horn do not integrate information arising from Aδ fibers activated by noxious heat. To further support this idea, it is long been known that the «wind-up» response is a specific property of C fibers in physiological conditions.^16^ In our experimental conditions, the sustained noxious hot stimulation protocol induced an immediate and short-lasting facilitation before progressively decreasing to a lower frequency of occurrence. This initial facilitation, absent in sustained cold stimulation, is likely to represent a truncated form of the «wind-up–like» phenomenon, ie, seen fully with electrical stimulation bypassing the natural detector of noxious heat.^19^ TRPV1-expressing sensory neurons may perfectly be responsible for this phenomenon because early facilitation may encounter the optimal activation of TRPV1 before its partial deactivation as previously described in the literature.^25^ Our results are in accordance with the recent study of Ran et al., who recorded in vivo the calcium activity of thermoresponsive spinal neurons after peripheral cutaneous stimulation.^31^ They showed that the integration of cold information is more relative than adaptative and does not depend on absolute temperature, whereas the integration of hot stimulation does and shows little to no adaptation. These results are perfectly in line with ours as illustrated in the hot and cold response curve supporting the existence of «broadly tuned» spinal neurons that are responsive to both hot and cold stimulations. This neuronal population represented most of the recorded WDR neurons in vivo.

In this study, we finely characterized the integration of thermal nociceptive stimuli by WDR neurons in the deep dorsal horn layers of the rat spinal cord. Our results claim for distinct local spinal circuits of convergence for hot and cold nociceptive stimuli, using C-type and A-type sensory afferents, respectively. This observation is of utmost interest for further research work analyzing pathological pain states where pain symptoms related to cold and hot stimuli are differentially expressed (eg, inflammatory vs neuropathic pain).

## Disclosures

The authors have no conflicts of interest to declare.

## Appendix A. Supplemental digital content

Supplemental digital content associated with this article can be found online at http://links.lww.com/PR9/A144.

## Supplementary Material

SUPPLEMENTARY MATERIAL

## References

[1] Abbadie C Honore P Besson JM. Intense cold noxious stimulation of the rat hindpaw induces c-fos expression in lumbar spinal cord neurons. Neuroscience 1994;59:457–68. 8008200 10.1016/0306-4522(94)90609-2

[2] Basbaum AI Bautista DM Scherrer G Julius D. Cellular and molecular mechanisms of pain. Cell 2009;139:267–84. 19837031 10.1016/j.cell.2009.09.028 PMC2852643

[3] Benoist JM Pincede I Ballantyne K Plaghki L Le Bars D. Peripheral and central determinants of a nociceptive reaction: an approach to psychophysics in the rat. PLoS One 2008;3:e3125. 18769624 10.1371/journal.pone.0003125 PMC2518957

[4] Campbell JN LaMotte RH. Latency to detection of first pain. Brain Res 1983;266:203–8. 6871657 10.1016/0006-8993(83)90650-9

[5] Castillo K Diaz-Franulic I Canan J Gonzalez-Nilo F Latorre R. Thermally activated TRP channels: molecular sensors for temperature detection. Phys Biol 2018;15:021001. 29135465 10.1088/1478-3975/aa9a6f

[6] Caterina MJ Schumacher MA Tominaga M Rosen TA Levine JD Julius D. The capsaicin receptor: a heat-activated ion channel in the pain pathway. Nature 1997;389:816–24. 9349813 10.1038/39807

[7] Dado RJ Katter JT Giesler GJ Jr. Spinothalamic and spinohypothalamic tract neurons in the cervical enlargement of rats. II. Responses to innocuous and noxious mechanical and thermal stimuli. J Neurophysiol 1994;71:981–1002. 8201437 10.1152/jn.1994.71.3.981

[8] Danneman PJ Kiritsy-Roy JA Morrow TJ Casey KL. Central delay of the laser-activated rat tail-flick reflex. PAIN 1994;58:39–44. 7970838 10.1016/0304-3959(94)90183-X

[9] Darian-Smith I Johnson KO Dykes R. Cold fiber population innervating palmar and digital skin of the monkey: responses to cooling pulses. J Neurophysiol 1973;36:325–46. 4196271 10.1152/jn.1973.36.2.325

[10] Davis KD Pope GE. Noxious cold evokes multiple sensations with distinct time courses. PAIN 2002;98:179–85. 12098630 10.1016/s0304-3959(02)00043-x

[11] Despres O Lithfous S Pebayle T Casadio C Dufour A. Effects of thermosensory aging well demonstrated by cold stimulations with high temporal resolution. Muscle Nerve 2019;60:141–6. 30945307 10.1002/mus.26482

[12] Dhaka A Viswanath V Patapoutian A. Trp ion channels and temperature sensation. Annu Rev Neurosci 2006;29:135–61. 16776582 10.1146/annurev.neuro.29.051605.112958

[13] Dubin AE Patapoutian A. Nociceptors: the sensors of the pain pathway. J Clin Invest 2010;120:3760–72. 21041958 10.1172/JCI42843 PMC2964977

[14] Dufour A Despres O Pebayle T Lithfous S. Thermal sensitivity in humans at the depth of thermal receptor endings beneath the skin: validation of a heat transfer model of the skin using high-temporal resolution stimuli. Eur J Appl Physiol 2020;120:1509–18. 32361772 10.1007/s00421-020-04372-y

[15] Filingeri D. Neurophysiology of skin thermal sensations. Compr Physiol 2016;6:1429. 27347898 10.1002/cphy.c150040

[16] Gozariu M Bragard D Willer JC Le Bars D. Temporal summation of C-fiber afferent inputs: competition between facilitatory and inhibitory effects on C-fiber reflex in the rat. J Neurophysiol 1997;78:3165–79. 9405536 10.1152/jn.1997.78.6.3165

[17] Granovsky Y Matre D Sokolik A Lorenz J Casey KL. Thermoreceptive innervation of human glabrous and hairy skin: a contact heat evoked potential analysis. PAIN 2005;115:238–47. 15911150 10.1016/j.pain.2005.02.017

[18] Han ZS Zhang ET Craig AD. Nociceptive and thermoreceptive lamina I neurons are anatomically distinct. Nat Neurosci 1998;1:218–25. 10195146 10.1038/665

[19] Herrero JF Laird JM Lopez-Garcia JA. Wind-up of spinal cord neurones and pain sensation: much ado about something? Prog Neurobiol 2000;61:169–203. 10704997 10.1016/s0301-0082(99)00051-9

[20] Huang SM Li X Yu Y Wang J Caterina MJ. TRPV3 and TRPV4 ion channels are not major contributors to mouse heat sensation. Mol Pain 2011;7:37. 21586160 10.1186/1744-8069-7-37 PMC3123222

[21] Hughes DI Todd AJ. Central nervous system targets: inhibitory interneurons in the spinal cord. Neurotherapeutics 2020;17:874–85. 33029722 10.1007/s13311-020-00936-0 PMC7641291

[22] Juif PE Poisbeau P. Neurohormonal effects of oxytocin and vasopressin receptor agonists on spinal pain processing in male rats. PAIN 2013;154:1449–56. 23707282 10.1016/j.pain.2013.05.003

[23] Khasabov SG Cain DM Thong D Mantyh PW Simone DA. Enhanced responses of spinal dorsal horn neurons to heat and cold stimuli following mild freeze injury to the skin. J Neurophysiol 2001;86:986–96. 11495966 10.1152/jn.2001.86.2.986

[24] Latorre R Brauchi S Orta G Zaelzer C Vargas G. ThermoTRP channels as modular proteins with allosteric gating. Cell Calcium 2007;42:427–38. 17499848 10.1016/j.ceca.2007.04.004

[25] Luo L Wang Y Li B Xu L Kamau PM Zheng J Yang F Yang S Lai R. Molecular basis for heat desensitization of TRPV1 ion channels. Nat Commun 2019;10:2134. 31086183 10.1038/s41467-019-09965-6 PMC6513986

[26] MacIver MB Tanelian DL. Structural and functional specialization of A delta and C fiber free nerve endings innervating rabbit corneal epithelium. J Neurosci 1993;13:4511–24. 8410200 10.1523/JNEUROSCI.13-10-04511.1993 PMC6576377

[27] McKemy DD Neuhausser WM Julius D. Identification of a cold receptor reveals a general role for TRP channels in thermosensation. Nature 2002;416:52–8. 11882888 10.1038/nature719

[28] Mendell LM Wall PD. Responses of single dorsal cord cells to peripheral cutaneous unmyelinated fibres. Nature 1965;206:97–9. 14334366 10.1038/206097a0

[29] Menetrey D Chaouch A Besson JM. Responses of spinal cord dorsal horn neurones to non-noxious and noxious cutaneous temperature changes in the spinal rat. PAIN 1979;6:265–82. 460934 10.1016/0304-3959(79)90048-4

[30] Paricio-Montesinos R Schwaller F Udhayachandran A Rau F Walcher J Evangelista R Vriens J Voets T Poulet JFA Lewin GR. The sensory coding of warm perception. Neuron 2020;106:830–41 e833. 32208171 10.1016/j.neuron.2020.02.035 PMC7272120

[31] Ran C Hoon MA Chen X. The coding of cutaneous temperature in the spinal cord. Nat Neurosci 2016;19:1201–9. 27455110 10.1038/nn.4350 PMC5599125

[32] Simone DA Kajander KC. Excitation of rat cutaneous nociceptors by noxious cold. Neurosci Lett 1996;213:53–6. 8844711 10.1016/0304-3940(96)12838-x

[33] Story GM Peier AM Reeve AJ Eid SR Mosbacher J Hricik TR Earley TJ Hergarden AC Andersson DA Hwang SW McIntyre P Jegla T Bevan S Patapoutian A. ANKTM1, a TRP-like channel expressed in nociceptive neurons, is activated by cold temperatures. Cell 2003;112:819–29. 12654248 10.1016/s0092-8674(03)00158-2

[34] Todd AJ Spike RC Young S Puskar Z. Fos induction in lamina I projection neurons in response to noxious thermal stimuli. Neuroscience 2005;131:209–17. 15680704 10.1016/j.neuroscience.2004.11.001

[35] Urch CE Dickenson AH. In vivo single unit extracellular recordings from spinal cord neurones of rats. Brain Res Brain Res Protoc 2003;12:26–34. 12928042 10.1016/s1385-299x(03)00068-0

[36] Vandewauw I De Clercq K Mulier M Held K Pinto S Van Ranst N Segal A Voet T Vennekens R Zimmermann K Vriens J Voets T. A TRP channel trio mediates acute noxious heat sensing. Nature 2018;555:662–6. 29539642 10.1038/nature26137

[37] Weast RC. CRC handbook of chemistry and physics. Boca Raton, FL: CRC Press, 1988.

[38] Winter J. TRPV1 distribution and regulation. In: Turning up the Heat on Pain: TRPV1 Receptors in Pain and Inflammation. AB Malberg, KR Bley, eds. Springer, 2005. pp. 39–51.

[39] Xu F Lin M Lu TJ. Modeling skin thermal pain sensation: role of non-Fourier thermal behavior in transduction process of nociceptor. Comput Biol Med 2010;40:478–86. 20417502 10.1016/j.compbiomed.2010.03.002

[40] Zhou S Lithfous S Despres O Pebayle T Bi X Dufour A. Involvement of frontal functions in pain tolerance in aging: evidence from neuropsychological assessments and gamma-band oscillations. Front Aging Neurosci 2020;12:131. 32536860 10.3389/fnagi.2020.00131 PMC7266988

