# Supplementary material for: Spinal integration of hot and cold nociceptive stimuli by wide-dynamic-range neurons in anesthetized adult rats
Source: Pain Rep. 2021 Dec 16;6(4):e983. doi: 10.1097/PR9.0000000000000983 (PMC8687733; doi:10.1097/PR9.0000000000000983)

Supplementary Figure 1: Male and female action potential firing frequencies of WDR neurons in the dorsal horn of the spinal cord after hot (panel A,C) and cold stimulation (panel B). Panels A and B correspond to responses to a single thermal stimulus (52°C or 0°C, speed: 300°C/s, duration: 1000ms) whereas panel C shows results for a slow heating (3°C/s, duration 7.5s) to 52°C. Statistic: Student's t-test; ns: non significant.

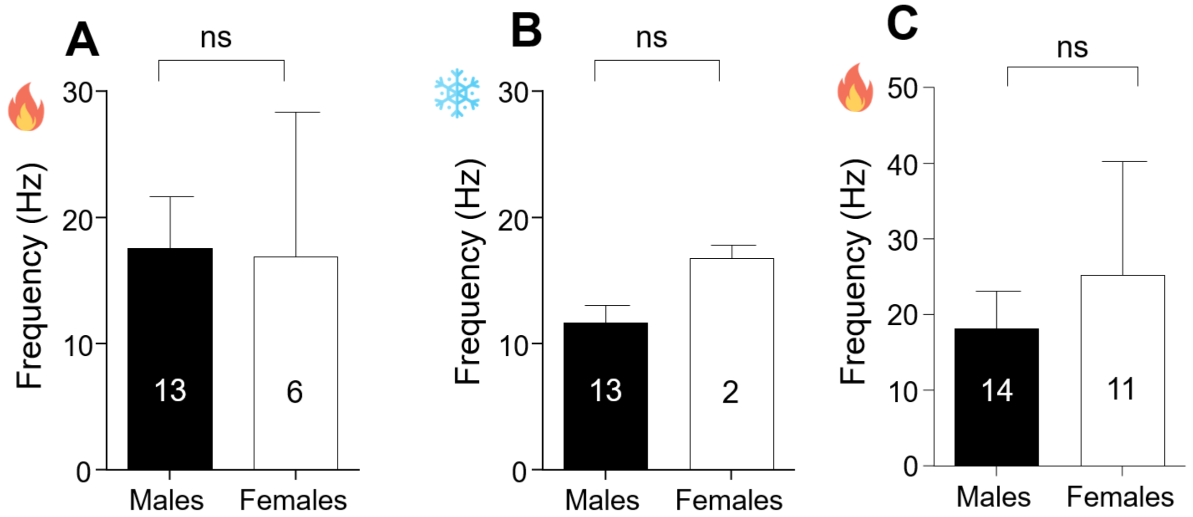

Supplement: SUPPLEMENTARY MATERIAL [file painreports-6-e983-s001.pdf]
